# Tracing thyroid hormone-disrupting compounds: database compilation and structure-activity evaluation for an effect-directed analysis of sediment

**DOI:** 10.1007/s00216-015-8736-9

**Published:** 2015-05-19

**Authors:** Jana M. Weiss, Patrik L. Andersson, Jin Zhang, Eszter Simon, Pim E. G. Leonards, Timo Hamers, Marja H. Lamoree

**Affiliations:** Institute for Environmental Studies (IVM), Faculty of Earth and Life Sciences, VU University, De Boelelaan 1087, 1081HV Amsterdam, The Netherlands; Chemistry Department, Umeå University, 901 87 Umeå, Sweden; Oekotoxzentrum, Eawag, BU F 15-19, Überlandstrasse 133, 8600 Dübendorf, Switzerland

**Keywords:** Thyroid hormone-disrupting compound (THDC), Transthyretin (TTR), Database, Structure-activity relationship (SAR), Effect-directed analysis (EDA), Sediment

## Abstract

A variety of anthropogenic compounds has been found to be capable of disrupting the endocrine systems of organisms, in laboratory studies as well as in wildlife. The most widely described endpoint is estrogenicity, but other hormonal disturbances, e.g., thyroid hormone disruption, are gaining more and more attention. Here, we present a review and chemical characterization, using principal component analysis, of organic compounds that have been tested for their capacity to bind competitively to the thyroid hormone transport protein transthyretin (TTR). The database contains 250 individual compounds and technical mixtures, of which 144 compounds are defined as TTR binders. Almost one third of these compounds (*n* = 52) were even more potent than the natural hormone thyroxine (T_4_). The database was used as a tool to assist in the identification of thyroid hormone-disrupting compounds (THDCs) in an effect-directed analysis (EDA) study of a sediment sample. Two compounds could be confirmed to contribute to the detected TTR-binding potency in the sediment sample, i.e., triclosan and nonylphenol technical mixture. They constituted less than 1 % of the TTR-binding potency of the unfractionated extract. The low rate of explained activity may be attributed to the challenges related to identification of unknown contaminants in combination with the limited knowledge about THDCs in general. This study demonstrates the need for databases containing compound-specific toxicological properties. In the framework of EDA, such a database could be used to assist in the identification and confirmation of causative compounds focusing on thyroid hormone disruption.

**Electronic supplementary material:**

The online version of this article (doi:10.1007/s00216-015-8736-9) contains supplementary material, which is available to authorized users.

## Introduction

Both anthropogenic and naturally occurring compounds that are widely spread in the environment have the potential to disrupt the endocrine system of organisms. Reported impacts of endocrine-disrupting compounds (EDCs) in wildlife are, e.g., decreased fertility, altered masculinity and femininity, compromised immune system, gross birth defects, and thyroid dysfunctions [1]. Thyroid hormones (TH) play an important role in the (embryonic) development and in the maintenance of a normal physiological state. Compounds in the environment that have TH-disrupting properties could have devastating effects on individuals as well as on whole populations. Therefore, it is important to identify thyroid hormone-disrupting compounds (THDCs) and to construct screening methodologies to be included in monitoring programs [2].

TH disruption can be caused through many different pathways, such as interference with TH metabolism, TH excretion, or TH transport [3–5]. In 2006, a review paper on TH disruption assays was published by the Organisation for Economic Co-operation and Development (OECD) [3], and in 2010, it was proposed to establish a thyroid scoping effort group (TSEG) to determine the state of in vitro thyroid assays since 2006. The purpose of the TSEG was to bring relevant in vitro thyroid assays to the attention of OECD member countries and provide recommendations for their development and use [2]. Out of the 18 assays reviewed, the top three of the selected assays focused on TH disruption via transport protein binding [3]. Available in vitro assays identifying THDCs on a mechanism-base have been thoroughly discussed in a recently published review [6]. This study also recommended using disrupted circulation and transport of TH via the binding to serum transport proteins for the evaluation of the TH-disrupting potency of compounds.

Thyroid hormones are lipophilic compounds that are poorly soluble in aqueous media, including blood plasma. Therefore, TH transport proteins that are present in the plasma are important as they increase the blood carrying capacity for TH. TH transport proteins are themselves regulated by TH; thus, compounds that change circulating levels of TH are also likely to alter the transport protein concentration which will further change the dynamics of this endocrine system [3, 6]. Vertebrates typically have three major TH transport proteins, i.e., transthyretin (TTR), thyroxine-binding globulin (TBG), and albumin. In general, these TH transport proteins have higher affinity for the precursor TH hormone thyroxine (T_4_), which is therefore considered as the transport form of TH, than for 3,3′,5-triiodothyronine (T_3_), which is the active form of TH after deiodination of T_4_. Although mammalian TTR indeed has higher affinity for T_4_, TTR from teleost fish, amphibians, reptiles, and birds has higher affinity for T_3_ than for T_4_ [7]. Also, the relative importance of the three TH transporter proteins differs per species. Whereas TBG carries about 75 % of the TH in humans, TTR is the most important TH-transporting protein in rodents, tadpoles, and fish. Albumin is a nonselective transport protein for hormones. For lower vertebrates, albumin has been suggested to be important for T_4_ transport, and TTR for T_3_ transport [8]. Binding of xenobiotics to TH-carrying proteins is important not only because it may disrupt TH transport but also because the proteins may carry the xenobiotics to TH target tissues. This is especially the case for TTR, which can cross the placenta and the blood-brain barrier [9].

Examples of compounds that are known to interfere with TH transport proteins are hydroxylated polychlorinated biphenyls (OH-PCBs) and hydroxylated polybrominated diphenylethers (OH-PBDEs) [4, 10, 11]. More recently, perfluorinated compounds were reported to bind to TTR [12]. By competing with the natural ligand, TTR binders prevent proper transport of TH and cause an increase in unbound TH which is then available for further metabolization and excretion. Several different in vitro assays have described the determination of the binding potency of environmental pollutants to TTR, either based on radiolabeled ligand competitive binding (RLBA) or on the use of non-radiolabeled ligand competitive binding (BA, SPR, ANSA, and FLU-TTR). The assays are described further in detail in the Electronic Supplementary Material.

To identify TH-disrupting compounds in the environment, the TTR-binding assay can be used in effect-directed analysis (EDA). EDA involves a bioassay/effect-directed fractionation of an extract followed by chemical identification and confirmation steps. EDA was successfully applied to evaluate endocrine potencies in several water systems, e.g., in wastewater treatment plants [13–15], rivers [13, 15–17], harbor areas [18, 19], marine sediments [20], and biota [21]. An earlier published review describes a range of important results from effect-directed studies, e.g., identified compounds and pinpointed hotspots [22]. Until now the main focus in EDA research has been on estrogenic and (anti-)androgenic activity and only four studies have addressed thyroid hormone disruption, two in sediment extracts from water systems, i.e., in The Netherlands [19] and in Germany [23], one in indoor dust from Japan [24], and one in polar bear plasma [25]. In polar bear plasma, the identified THDCs could successfully explain almost 80 % of the observed TTR-binding potency of the extract.

A major criticism of the EDA approach is the limited identification success rate, and a key to increase its utility is to improve the availability and quality of databases containing known bioactive compounds and their chemical properties. To aid the identification of effect-causing compounds, we compiled a database that reports all compounds that have been tested in the TTR-binding assay, including both active and non-active compounds. To further explore the chemical characteristics of TTR-binding compounds, the compiled dataset was analyzed using principal component analysis (PCA) and a set of calculated chemical descriptors.

A second aim of the paper was to use the database to assist in the identification of THDCs in a sediment extract using the EDA approach [26].

## Methods

### Database of compounds tested for their TTR-binding potency

A database was compiled with compounds that were tested in in vitro bioassays measuring the TTR-binding potency (Table S1). The in vitro bioassays are described in the Electronic Supplementary Material. The literature was collected via ScienceDirect and PubMed, and the search was limited to compounds tested for having a direct interaction with TTR. The relative potencies, expressed in T_4_ or T_3_ relative effect potency (REP) values, were either extracted from the publications or calculated by dividing the IC_50_ of the natural ligand T_4_ by the IC_50_ of the competitor, with IC_50_ as the concentration that is capable of replacing 50 % of a labeled TTR ligand from TTR. By definition, the REP of T_4_ and T_3_ is 1. Thus, compounds with higher affinity for TTR than the natural ligand have a REP > 1, and compounds with lower affinity have a REP < 1.

### Chemical descriptors and principal component analysis

Of the 250 compounds in the database, 220 have been tested for their binding affinity to human TTR. These compounds were classified as binders (IC_50_ > 25 μM; 100 compounds) and non-binders (IC_50_ > 25 μM; 120 compounds) and characterized using 57 chemical descriptors calculated using the Molecular Operating Environment (MOE; chemcomp.com) software from their structures (represented by SMILES codes). The structures of the compounds were taken from various sources (e.g., SciFinder, PubChem) and processed by ChemAxon JChem Standardizer under the settings of “Add explicit hydrogens,” “Aromatize,” “Clean 2D,” and “Remove fragment” (ChemAxon JChem Version 6.1.2, 2013. ChemAxon; chemaxon.com). Descriptors were selected for their chemical relevance, interpretability, and (hence) utility for describing their major chemical variation. A complete list with brief explanations can be found in the Electronic Supplementary Material (Table S2). The descriptor set includes the logarithm of the octanol-water partition coefficient (log *K*_ow_), molecular polarizability, and Van der Waals volume in combination with selected flexibility, shape, and connectivity indices. In addition, molecular surface characteristics were reflected by “partial equalization or orbital electronegativity” (PEOE) descriptors. Counts of selected atom types, single and aromatic bonds, and some count ratios completed the descriptor set.

Prior to the analysis, the descriptors were logarithmically transformed (if not already log transformed) if the calculated skewness exceeded 2 to improve the normality of the descriptors’ distributions and to minimize the impact of extreme values.

PCA was used to obtain an overview of the physicochemical characteristics of the compounds of the database. PCA is a latent vector-based method that compresses data into a few orthogonal vectors, principal components (PCs), summarizing the variation and correlation patterns in the data. Each PC consists of two vectors where not only similarities among objects (i.e., compounds) but also correlation patterns among the descriptors are visualized. The score vector shows similarities among the compounds, and the loading vector shows the correlation pattern among the descriptors. Here, we used the software SIMCA-P+ v13 for the multivariate analysis (umetrics.com) and an eigenvalue of 2 to define the number of significant PCs.

### Chemical screening of TTR binders in sediment

The created TTR-binding database was used to identify THDC in a sediment sample from a previous EDA study. The sediment sample was collected from a polluted site in the river Schijn, a tributary to the river Scheldt, close to Antwerp (Belgium). The sample, EDA methodology, and chemical analysis have been described in detail elsewhere [16, 26].

In short, the sample preparation included accelerated solvent extraction, gel permeation chromatography, and fractionation by reversed-phase (RP) followed by normal-phase (NP) liquid chromatography techniques. Each RP and NP fraction was tested for its TTR-binding potency in the RLBA, described in detail elsewhere [4, 12]. The assay has been validated in-house, showing a coefficient of variation of less than 8 % over time. The limit of detection is set to 20 % of the binding capacity (in the range of 16 nM T_4_). To improve the detection limit, the ^125^I-T_4_ is always purified from free labeled iodine before incubation [12].

The identification of active compounds is facilitated by a decrease in complexity of the sample extract, which is obtained by repeated fractionation of samples with TTR-binding activity. Nevertheless, still many compounds showed to be present in the active fractions. All NP fractions were screened with GC-MS (GC Agilent 6890 with a MS, Agilent 5973), equipped with an SGE-BPX5 column (25 m, 0.22 mm i.d., 0.3 μm film thickness), using splitless injection and full-scan (*m*/*z* 50–650) data acquisition in electron impact (EI) mode [26], and with LC-MS using a linear quadrupole ion trap-Orbitrap (LTQ-Orbitrap) mass spectrometer (Thermo Electron) with an Xbridge C18-LC column (Waters, 100 × 2.1 mm, 3.5 μm) equipped with an electron spray ionization source (ESI) operating in positive mode (negative mode was evaluated but is not reported due to lack of interesting results) [16]. Survey full-scan MS spectra (from *m*/*z* 50 to 600) were acquired using the Orbitrap with a resolution of 30,000.

GC-MS spectra were deconvoluted using the Automated Mass Spectral Deconvolution and Identification System (AMDIS version 2.64) and compared with reference spectra in the National Institute of Standards and Technology (NIST version 02) main mass spectral database (match factor ≥70 %) for tentative identification [26]. The identification using LC coupled to high-resolution LTQ-Orbitrap instrumentation was described in detail elsewhere [16].

## Results and discussion

### Chemical characterization of TTR binders

In total, 427 entries representing 250 compounds and technical mixtures were included in the database. All compounds have been tested for their binding potency to TTR, derived from seven different species, human (recombinant and purified, 305 entries), birds (chicken [*Gallus gallus*] and gull [*Larus hyperboreus* and *Larus argentatus*], 26 entries), fishes (salmon [*Onchorhynchus masou*] and sea bream, 51 entries), and amphibians (*Rana catesbeiana* and *Xenopus laevis*, 45 entries).

**Fig. 1:**
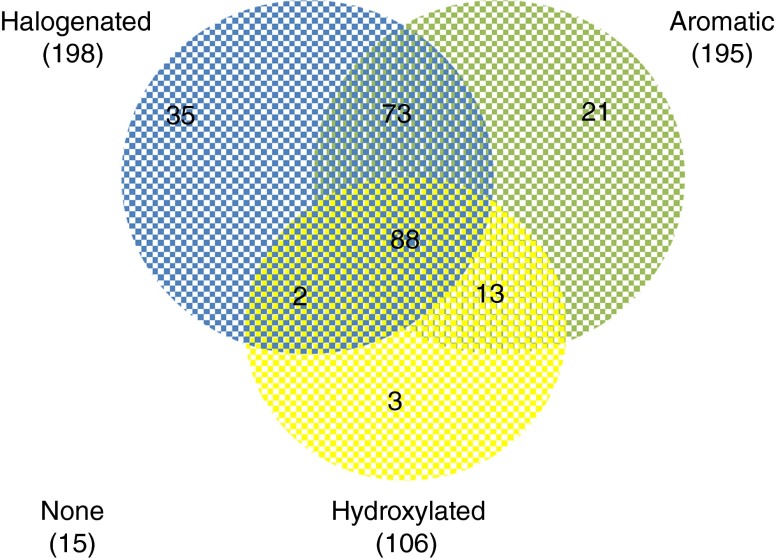
A Venn diagram illustrating the distribution of the three main chemical structure characteristics, i.e., halogenation, aromaticity, and the presence of a hydroxyl group, of the 250 compounds tested in the TTR-binding assays listed in Table S1 (Electronic Supplementary Material). In *brackets* are the total numbers of compounds belonging to each characteristic or to none of the three characteristics

Among the 250 compounds of the database, 222 compounds were derived from the literature and 33 compounds were tested in the present study, of which 5 have been analyzed earlier for their potency to bind to TTR. The distribution of the most common characteristics of the 250 entities is illustrated in a Venn diagram (Fig. 1); 198 compounds are halogenated (79 %); 195 contain ≥1 aromatic ring system (78 %); 106 compounds are hydroxylated (42 %); 88 compounds are aromatic, halogenated, and hydoxylated (35 %); and 15 (6 %) of the compounds lack the three main characteristics, i.e., aromatic, hydroxylated, and halogenated. Based on knowledge of the structure of known environmental THDCs, halogenated aryl compounds, with a hydroxy group at a position similar to the natural ligand thyroxine (T_4_), are traditionally suspected to have TTR-binding potencies. Compounds with carboxylic acids were not considered as hydroxylated because these acids are most likely not protonated under natural conditions. One hundred forty-four of the tested compounds (58 %) were able to bind to TTR, and 52 (21 %) were more potent than the natural hormone T_4_ (REP > 1). Among the 52 most potent compounds, 48 were aromatic, hydroxylated, and halogenated.

Some differences in the TTR-binding potency between species have been reported [27, 28] (see examples in Table S1). Although the primary structure of TTR has been conserved for 400 million years, some changes in amino acid residues of the N-terminal region have been identified [29]. These structural modifications affect the affinity of the TTR-binding site not only to T_4_ but also to exogenous compounds, and explain the differences in the TTR-binding potency of compounds reported between species.

**Fig. 2:**
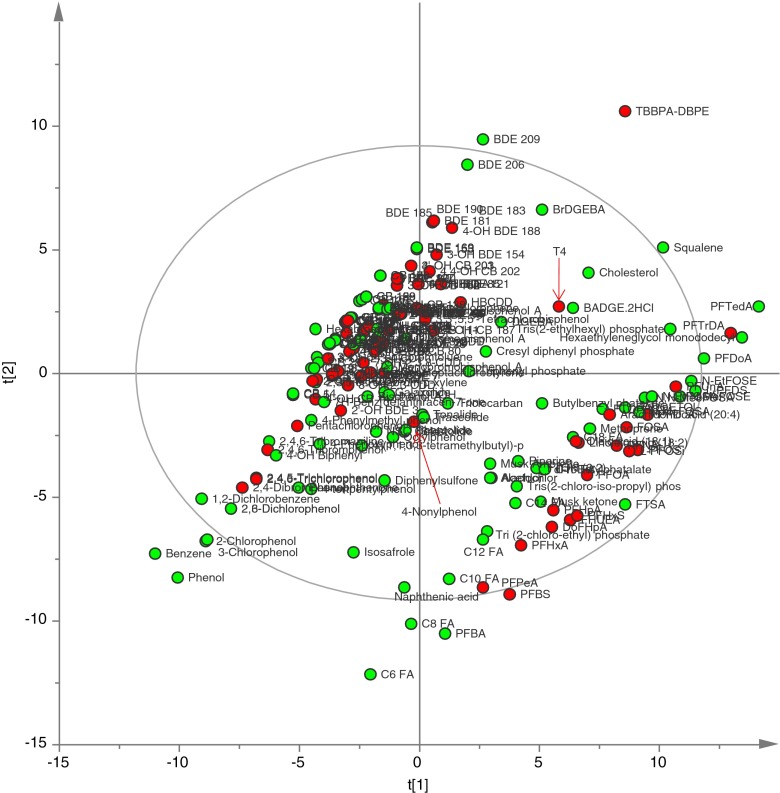
PCA score plot showing principal component 1 (t1) versus 2 (t2). Compounds marked in *red* are reported with IC_50_ values ≤25 μM and in *green* >25 μM in the human TTR assay. Compounds are abbreviated according to Table S1

A PCA was applied to view the chemical variation of the 220 compounds tested for their binding affinity to human TTR. Four significant principal components (PCs) could explain 85 % of the chemical variation, of which the first two PCs explained 64 % (Fig. 2—score plot). In brief, the two first PCs display a separation into three groups covering aromatic and halogenated compounds (group 1, consisting of binders and non-binders), fatty acids (group 2, non-binders), and perfluorinated compounds (group 3, binders and non-binders). Each group is spread in the second dimension (PC2) according to polarity and molecular size (Figure S1—loading plot). The majority of active compounds is clustered (the left cluster), and these have negative PC1 values and moderate PC2 values. In this cluster, the small phenols are inactive as well as the large brominated aromatics (Fig. 2). The PCA clearly illustrates the disparate chemical properties of the perfluorinated compounds (the right cluster) in relation to the majority of active compounds which indicates dissimilar structure-activity relationships. Noteworthy are also the unique chemical profiles of the endogenous hormone (T_4_) and the TTR binders tetrabromobisphenol A bis(2*,*3*-*dibromopropyl ether) TBBPA-DBPE and 4-nonylphenol (technical mixture). The reported activity of TBBPA-DBPE could, however, be due to contamination with its potent precursor TBBPA [30].

### Screening of THDCs in a sediment sample

#### TTR-binding activity in the sediment extract

**Fig. 3:**
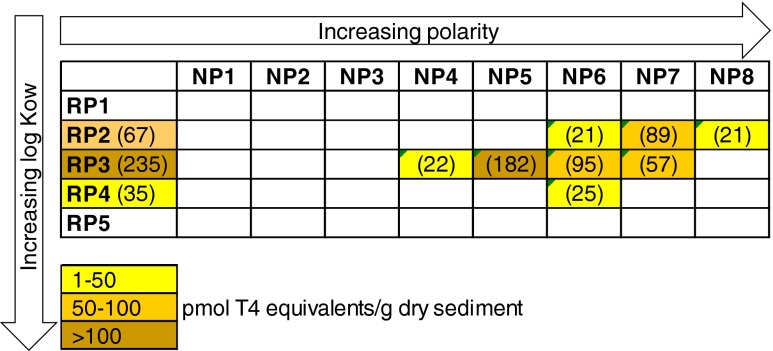
The TTR-binding activity measured in the reversed-phase (RP) fractions and the normal-phase (NP) fractions. The activity (pmol T_4_ equivalents/g dry sediment) is given in *brackets* for each fraction

An EDA study of sediment was conducted to determine the TTR-binding potency and to identify the active compounds. Before the sediment extract was fractionated, the extract showed a total TTR-binding potency of 1000 pmol T_4_ equivalents/g dry weight (d.w.) of sediment. Using reversed-phase (RP) LC, the extract was fractionated into five fractions with an increasing octanol-water partitioning coefficient (*K*_ow_) (RP1–RP5), of which RP3 showed the highest TTR-binding affinity (i.e., 235 pmol T_4_ equivalents/g d.w.; first column in Fig. 3). Active RP fractions were further fractionated by normal-phase (NP) LC into eight fractions with increasing polarity. For the active RP3 fraction, this resulted in three active NP fractions, i.e., NP 5–7. Elevated TTR-binding potency was also found in RP2NP7. The summarized TTR-binding potency of the individual fractions was ca 50 % of the total TTR-binding potency measured in the unfractionated extract. This lower value may be explained by the separation of active compound groups over multiple fractions, resulting in lower potencies of the individual fractions close to the limit of quantification of the assay. The recovery of the potency of TTR binding was tested by pooling the NP fractions into the original composition and performing the assay again. It was confirmed that the decrease in potency after fractionation was not due to loss of compounds in the fractionation step as the initial potency was recovered in the pooled NP fractions (data not shown).

#### Identification of THDCs in active fractions

To identify which compounds were present in the active fractions, LTQ-Orbitrap and GC-MS analyses were carried out, from which 43 tentatively identified compounds were derived (Table 1). The LTQ-Orbitrap results were evaluated using the identification strategy described earlier [16]. From a total number of 6503 base peak framed masses (SIEVE), 337 accurate masses were selected for investigation. Criteria were that the peak shape was normally distributed and the mass should have an intensity 100 times higher than the adjacent control (non-active) fraction. It was not possible to perform a mass fragmentation confirmation to establish the match factor of the tentatively identified compounds in the LTQ-Orbitrap due to low intensities. Instead, the accurate mass and the isotope pattern were used for tentative identification. With this strategy, eight compounds could be tentatively identified which were present in the NIST library and then analytically confirmed by retention time using standard compounds (marked LC in Table 1).

**Table 1:** Tentatively identified compounds (analyzed with GC-MS with a match factor ≥70 % in the NIST library, or with LC-LTQ-Orbitrap on accurate mass) present in TTR-binding active fractions (RP = reversed-phase and NP = normal-phase fractionation) in a sediment sample. The span of the log *K*_ow_ of the RP fractions RP2, RP3, and RP4 is in the order of 2–4, 4–6, and 6–9 respectively. In addition, eight compounds indicated in the NIST library with a match factor of 50–70 % and the additionally tested compounds in the TTR-binding assay are listed at the end of the table

Fraction	CAS	Name	LC/GC	TTR-binding activity
RP2NP6	123-52-85	(1*H*-Benzoimidazol-2-yl)-diphenyl-methanol	GC	No info
	4237-44-9	2-(1-Phenylethyl)-phenol	GC	No info
	23966-56-5	2-Amino-3,3-diphenyl-phthalimidine	GC	No info
	NIST-307688	4-Methoxyphenyl ester 2-fluorobenzoic acid	GC	No info
	85-68-7	Butylbenzyl phthalate	GC	Active [28], not active (TS)
	127-63-9	Diphenyl sulfone	GC	Not active (TS)
RP2NP7	137909-40-1	Bis(1-chloro-2-propyl)(3-chloro-1-propyl) phosphate	GC	No info
	115-86-6	Triphenyl phosphate	GC	Not active (TS)
	13674-84-5	Tris(2-chloro-iso-propyl) phosphate	GC	Not active (TS)
	1067-98-7	Tris(3-chloropropyl) phosphate	GC	No info
	120-58-1	Isosafrole	LC	Not active (TS)
	548-39-0	Perinaphthenone	LC/GC	Not active (TS)
	959-28-4	(2*E*)-1,4-Diphenyl-2-butene-1,4-dione	LC	Not active (TS)
	94-62-2	Piperine	LC	Not active (TS)
RP3NP4	225-51-4	Benz[c]acridine	GC	No info
	1018-97-9	Bis(2-methylphenyl)-methanone	GC	No info
	1222-05-5	Galaxolide	LC/GC	Not active (TS)
RP3NP5	1159-86-0	1,2-Dibenzoyl benzene	GC	No info
	3770-82-9	1,3-Dibenzoyl benzene	GC	No info
	3016-97-5	1,4-Dibenzoylbenzene	GC	No info
	75694-46-1	2,3-Diphenylbenzo-1,4-dioxin	GC	No info
	2219-84-3	2-Methyl-4-(1,1,3,3-tetramethylbutyl)-phenol	GC	Not active (TS)
	140-66-9	4-(1,1,3,3-Tetramethylbutyl)-phenol	GC	Not active (TS)
	80-46-6	4-*tert-*Pentyl phenol	GC	Not active (TS)
	5635-50-7	Hexestrol	GC	No info
	21145-77-7	Tonalide	LC/GC	Not active (TS)
	68140-48-7	Traseolide	LC/GC	Not active (TS)
RP3NP5-6	82-05-3	7*H*-Benz[*de*]anthracen-7-one^a^	LC	Not active (TS)
	NIST-317319	*n*-[4-(Phenylamino)phenyl]-benzamide	GC	No info
	104-40-5	*p*-Nonylphenol technical mixture, 8 identified peaks	GC	Active (TS and [40, 25])
RP3NP6	20760-63-8	1-(*p*-Fluorophenyl)-anthraquinone	GC	No info
	NIST-307638	3,4-Dichlorophenyl ester *p*-anisic acid	GC	No info
	64436-60-8	4′-Propoxy-2-methylpropiophenone	GC	No info
	NIST-315373	Di(3-methylphenyl) phthalate	GC	No info
	84-74-2	Di-*n*-butyl phthalate	GC	Active [28], not active (TS)
	NIST-315190	Ethyl hex-2-yn-4-yl phthalate	GC	No info
	13556-73-5	*n*-Benzyl-*n*′-phenyl-*p*-phenylenediamine	GC	No info
	3380-34-5	Triclosan	GC	Active (TS)
RP3NP7	5875-45-6	2,5-Bis(1,1-dimethylethyl)-phenol	GC	No info
RP4NP6	80-97-7	Cholestanol	GC	No info
	360-68-9	Coprostanol	GC	No info
	559-74-0	Friedelan-3-one	GC	No info
	1617-70-5	Lupenone	GC	No info
Compounds indicated in the NIST library (match factor 50–70 %)				
Multiple	101-53-1	4-(Phenylmethyl)-phenol	GC	Not active (TS)
Multiple	1806-26-4	4-Octylphenol	GC	Active [28], not active (TS)
Multiple	26444-49-5	Cresyldiphenyl phosphate	GC	Not active (TS)
Multiple	3055-96-7	Hexaethyleneglycol monododecyl ether	GC	Not active (TS)
Multiple	1338-24-5	Naphthenic acid	GC	Not active (TS)
Multiple	111-02-4	Squalene	GC	Not active (TS)
Multiple	115-96-8	Tris(2-chloro-ethyl) phosphate	GC	Not active (TS)
Multiple	78-42-2	Tris(2-ethylhexyl) phosphate	GC	Not active (TS)
Additional tested compounds (not tentatively identified in the sediment sample)				
ᅟ	80-05-7	Bisphenol A		Not active (TS and [5])
ᅟ	13171-00-1	Celestolide		Not active (TS)
ᅟ	15323-35-0	Phantolide		Not active (TS)
ᅟ	83-66-9	Musk ambrette		Not active (TS)
ᅟ	81-14-1	Musk ketone		Not active (TS)
ᅟ	101-20-2	Triclocarban		Not active (TS)
ᅟ	15307-86-5	Diclofenac		Active (TS)

*No info* no available literature on TTR-binding activity and not tested in this study, *TS* this study

GC-MS screening using AMDIS and the NIST library resulted in the tentative identification of 39 compounds having a >70 % match factor (marked GC in Table 1). As this was a complex matrix, mass shifts and interfering effects such as ion suppression may have occurred, possibly altering the chromatographic profile. Therefore, also compounds suggested from the NIST library search with a match factor below 70 % could be of interest. Eight NIST library search indicated compounds, with a match factor between 50 and 70 %, are listed in Table 1, which were available in-house and tested for their TTR-binding potency.

Out of the tentatively identified compounds in the sediment sample, only two could be confirmed as TTR binders, i.e., triclosan and the technical mixture of nonylphenol. Triclosan is an antibacterial agent, which has shown to cause a dose-dependent decrease in total T_4_ in a 4-day oral exposure study with rats [31]. TTR binding could be an explanation for this observed decrease of free circulating T_4_. Nonylphenol is a detergent, which is used in a technical mixture and also a degradation product of nonylphenol polyethoxylate surfactants. Eight different isomers were distinguished in the GC chromatogram, consisting of linear and differently branched isoforms typically present in a technical mixture. An earlier study confirmed that branched nonylphenol can bind to human TTR, whereas no binding was observed for linear nonylphenol [25]. Nonylphenol has been reported before as a binder to TTR from several species (Table S1), but these studies did not report which form of nonylphenol was tested. Earlier analysis of exactly the same sample reported a triclosan concentration of 26 ng/g d.w. sediment and a nonylphenol concentration of 210 ng/g d.w. [32]. Based on their REP values and concentrations, these two compounds could explain less than 1 % of the observed TTR-binding potency in the unfractionated extract. Only four of the compounds in Table 1 have been studied earlier for their TTR-binding potency, i.e., the nonylphenol, two phthalates (butylbenzyl phthalate and dibutyl phthalate), and octylphenol. The phthalates and octylphenol were reported to exhibit weak TTR binding in RLBA with salmon and frog TTR [28] (Table S1). In the present study, however, these compounds showed no binding to human TTR. The four compounds present in fraction RP4NP6 are a group of natural compounds including plant steroids (Table 1). Although the log *K*_ow_ values are slightly too high (~8.8) for the corresponding fraction (log *K*_ow_ 6–8), it is likely that these compounds are present in the sediment extract. The compounds are more likely to have an androgen- or estrogen-disrupting potential than TTR-binding activity, considering the structural similarities with steroid hormones.

In addition, the last seven compounds included in Table 1 were tested for their TTR-binding potency, due to their similar properties (aromatic, halogenated, and hydroxylated) as already tested compounds. Bisphenol A is a high-production-volume chemical (HPVC) used in plastics and epoxy resins, with high concern for its endocrine-disrupting properties, though no TTR-binding activity was observed. The musk compounds, celestolide, phantolide, ambrette, and ketone, did not bind to TTR. The antiseptic agent triclocarban is a common additive in soaps and other personal care products, and also for triclocarban, no TTR-binding activity was found.

Diclofenac is a non-steroidal anti-inflammatory drug (NSAID) used at high volume and is known to pass through the wastewater treatment plants and enter the water environment [33]. Diclofenac bound to TTR with a T_4_ REP value of 0.032 (Table S1). Diclofenac is also known for its binding potency to TTR from earlier studies on NSAIDs as anti-amyloid compounds [34]. Anti-amyloid genesis drugs are small molecules that stabilize TTR by binding to its TH binding sites (based on an in vitro fibril formation assay). Diclofenac is aromatic and chlorinated in ortho-positions and has a carboxylic acid moiety on the second aromatic ring. The binding pocket of TTR is well defined, and the size and shape of the ligand are important for the affinity [35]. It has been shown that biphenyls, dibenzofurans, diphenylethers, stilbene, and flavone are basic structures with optimal size that can be functionalized with substituents to interact in the pocket [36]. The compounds listed in Table 1 fit well into that description. Of the 58 compounds in Table 1, 10 compounds are halogenated (17 %), 45 have aromatic ring systems (76 %), and 15 are hydroxylated (26 %).

#### EDA strategy

The identification strategy presented here is limited by several set criteria (e.g., the intensity ratio active/non-active control fraction >100, match factor >70 %, standard availability, etc.). Another approach that currently receives significant attention is “suspect screening” using databases such as the one presented here. This strategy screens all masses but only the hits in the database are being further evaluated, which is an essential difference in comparison with the non-target screening strategy presented here. In addition, the prerequisite for the compound to be present in the NIST library limits the possibilities to identify active compounds which are metabolites and at low concentrations. Hence, future research is needed to aid the identification of the key toxicants present in the environment by developing more sensitive strategies for the chemical identification in the framework of EDA. Most crucial is the capability to exclude the presence of background compounds without losing the compounds of interest. One promising approach is the application of high-resolution fractionation techniques [37] or the application of the two-dimensional separation with a LC × LC system [38]. This together can improve the EDA efficiency regarding throughput and success rate. Still, it is important to get a better understanding and overview of chemical structures and effect-based correlations to carry out EDA in the search for key toxicants in our environment. Hopefully, the database presented here is one step in the direction for improved THDC identification.

## Conclusion

Table S1 in the Electronic Supplementary Material summarizes the state of knowledge regarding the TTR-binding capacity of a wide range of chemical compounds, e.g., environmental contaminants, pesticides, pharmaceuticals, metabolites, etc. The table is presented to facilitate future EDA studies to identify TTR binders and non-binders. Here, we have demonstrated an identification strategy in identifying THDCs in a sediment extract with TTR-binding potency. Two compounds, triclosan and nonylphenol technical mixture, could be confirmed to have contributed to the observed activity in the TTR-binding assay; however, only 1 % of the activity could be explained by the presence of these two compounds. The chemical properties in Table S1 are biased towards aromatic, halogenated, and hydroxylated compounds, which are structural features of the natural hormone thyroxine. The PCA illustrated this in the first PCs which displayed a separation of aromatic and halogenated compounds (active and non-active) from fatty acids (non-active) and perfluorinated compounds (active). In addition, the database presented here was used to further evaluate the structure relationship with the TTR-binding activity and a model was developed to predict the potential of contaminants to bind to TTR [39]. Here, it is demonstrated that more data on TTR-binding compounds is needed to cover the potential binding potency of compounds without these typical characteristics. Also, more sensitive identification strategies combining, e.g., novel fractionation techniques and accurate chemical analysis instruments are needed, without increasing the already substantial workload on the identification step.

## Electronic supplementary material

ESM 1 (PDF 422 kb)
