# Supplementary material for: Tracing thyroid hormone-disrupting compounds: database compilation and structure-activity evaluation for an effect-directed analysis of sediment
Source: Anal Bioanal Chem. 2015 May 19;407(19):5625–34. doi: 10.1007/s00216-015-8736-9 (PMC4498237; doi:10.1007/s00216-015-8736-9)
Supplement: Supplementary file 1 — (PDF 422 kb) [file 216_2015_8736_MOESM1_ESM.pdf]

Analytical and Bioanalytical Chemistry

Electronic Supplementary Material

Tracing thyroid hormone-disrupting compounds: database compilation and structure-activity evaluation for an effect-directed analysis of sediment

Jana M. Weiss, Patrik L. Andersson, Jin Zhang, Eszter Simon,

Pim E.G. Leonards, Timo Hamers and Marja H. Lamoree

Available bioassays to measure disruption of thyroid hormone transport via transthyretin binding

In the literature, several *in vitro* assays have been reported for the determination of the potency of environmental pollutants to bind to TTR. The most common *in vitro* assay is the radioligand binding assay (RLBA), which is a competitive binding assay using T_4 or T_3 (native and labeled) in a mixture with the competitor (compounds or extracts) to bind to TTR. The specific parameters of the assay differ slightly between laboratories, such as incubation temperature and time, buffer solution and packing material of the column for separating bound from free (labeled) TH.

Another approach is the non-radioligand binding assays (BA) using covalently bound ligands to different material. Three different BA approaches are represented here:

1. The use of polyclonal TTR antibodies covalently bound to a sepharose resin that is mixed with plasma pretreated with interfering compounds, i.e. the thyroid hormone disrupting compounds (THDCs). The TTR-THDC complex is washed and finally the THDC is eluted with high pH solution and injected onto an HPLC column for analysis and quantification [1]. The antibody capture of the complex can underestimate the THDC binding stoichiometry, owing to the weak

association between TTR and THDC and hence the THDC can dissociate during the wash step.

2. The weak association between transport protein and ligand is a property used in the second assay, the surface plasmon resonance (SPR) assay. In the SPR assay the thyroid hormone (TH) is covalently bound to a gold-layered chip, and an incubation mixture of the transport protein and competitors (THDCs) are injected in a flow cell passing over the bound thyroid hormone layer [2]. The association TTR-THDC complex is not stable and there will be a competition in the flow cell where available TTR will bind to the bound TH and affect the surface of the gold-layered chip. The change of the surface layer composition is detected in real time with SPR technique.

3. The third non-radioligand binding assay is the FLU-TTR, based on a protein-binding fluorescence probe (8-anilino-1-naphthalenesulfonic acid ammonium salt – ANSA or fluorescein−thyroxine), which becomes highly fluorescent after its binding to protein [3,4]. If an analyte binds to the protein at the same site as the probe, it will displace the probe from the protein and reduce the fluorescence intensity.

The radioligand and non-radioligand assays give comparable results (Table S1) but it is still difficult to compare studies using different parameters in their test setup, such as the use of T_4 or T_3, and different sources of TTR. For instance, the use of TTR from human, bird, fish and amphibians has been reported, both in purified and recombinant forms which influences the structure of the ligand binding site and the affinity.

One of the most commonly used bioassay is the well-established radioligand TTR binding assay based on the method described by Somack and co-workers [5], but with several minor modifications between laboratories [6-8]. The assay successfully detected TTR-binding activity of several pure compounds [6,7,9] and was also implemented for sediment [10,11] and biota extracts [12]. To test the applicability of the TTR-binding assay in EDA studies we present here the results from a sediment sample extract with a high TTR-binding potency.

Table S1: Compounds that have been tested in the *in vitro* TTR-binding assays reported in the literature are summarized. The backbone structure of the compound groups are presented together with the number of aromatic rings (ar). The compound name, the assay type, species and source of TTR (recombinant [rTTR], purified [pTTR] or non-specified) are given. The TTR binding potency is given as the concentration at 50% inhibition (IC_{50}) for either T_3 or T_4 and the corresponding relative effect potency (REP). All study references are given in the last column

Back bone	Compound	Assay	TTR	T_4 IC_{50} (nM)	T_4 REP	T_3 IC_{50} (nM)	T_3 REP	Ref
Diphenyl ether, 2 ar	Triiodothyronine (T_3)	BA	Human TTR			280		[3]
		FLU-TTR	Human TTR			1472	0.16	[4]
		RLBA	Gull rTTR			6	1	[8]
		RLBA	Chicken pTTR			22.5	1	[13]
		RLBA	Rana pTTR			1.43	1	[13]
		RLBA	Chicken pTTR			21	1	[14]
		RLBA	Rana pTTR			0.5	1	[14]
		RLBA	SeabreamrTTR			24	0.9	[15]
		RLBA	Rana rTTR			9		[16]
		RLBA	Xenopus TTR			400-550		[16]
	Thyroxine (T_4)	RLBA	Human pTTR	60-80	1			TS
		SPR	Human pTTR		1			[2]
		SPR	Human rTTR		1		0.06	[2]
		BA	Human TTR	30				[3]
		FLU-TTR	Human TTR	260	1			[4]
		RLBA	Human pTTR	55	1			[6,17]
		RLBA	Human rTTR	88, 138	1			[7]
		RLBA	Human TTR	60				[9]
		RLBA	Human TTR	80.7	1			[18]
		RLBA	Human rTTR	57	1			[19]

Back bone	Compound	Assay	TTR	T_4 IC_{50} (nM)	T_4 REP	T_3 IC_{50} (nM)	T_3 REP	Ref
		RLBA	Human rTTR	81	1			[20]
		RLBA	Human rTTR	79				[21]
		RLBA	Human TTR	79				[22]
		RLBA	Human rTTR	75				[23]
		RLBA	Human TTR	400	1			[24]
		RLBA	Human TTR	62				[25]
		ANSA	Human TTR	260	1			[26]
		RLBA	Gull rTTR	16	1			[8]
		RLBA	Seabream rTTR	21	1			[15]
		RLBA	Rana rTTR	500-700				[16]
		RLBA	Xenopus TTR	>10000				[16]
Benzene, 1 ar	Benzene	RLBA	Human TTR	0^{c}				[24]
	Acetochlor	RLBA	Rana TTR			79^{b}		[14]
		RLBA	Salmon TTR			87.3^{b}		[14]
		RLBA	Human TTR	n.d.	n.d.			[25]
	Alachlor	RLBA	Human TTR	n.d.	n.d.			[25]
	1.2-Dichlorobenzene	RLBA	Human TTR	0^{c}				[24]
	2,4-Dichlorophenoxyacetic acid	RLBA	Rana TTR			99.7^{b}		[14]
		RLBA	Salmon TTR			91^{b}		[14]
	2,4,6-Tribromaniline	RLBA	Human rTTR	n.d.	n.d.			[20]
	2,3,5,6-Tetrabrom-p-xylene	RLBA	Human rTTR	n.d.	n.d.			[20]
	2,3,4,5,6-Pentabromtoluene	RLBA	Human rTTR	n.d.	n.d.			[20]
	Hexabrombenzene	RLBA	Human rTTR	n.d.	n.d.			[20]
	Ioxynil	RLBA	Fish rTTR	39	0.5			[15]
		RLBA	Rana pTTR			100	0.005	[14]

Back bone	Compound	Assay	TTR	T_4 IC_{50} (nM)	T_4 REP	T_3 IC_{50} (nM)	T_3 REP	Ref
		RLBA	Chicken pTTR			15	1.4	[14]
		RLBA	Rana TTR			25.8[b]		[14]
		RLBA	Salmon TTR			9.2[b]		[14]
	Phenol	RLBA	Human TTR	10[c]				[24]
	Octylphenol	RLBA	Human pTTR	n.d.	n.d.			TS
		RLBA	Rana TTR			84.2[b]		[14]
		RLBA	Salmon TTR			85.5[b]		[14]
	Nonylphenol	RLBA	Seabream rTTR	17040	0.001			[15]
	technical mix	RLBA	Human pTTR	10000[d]	0.008			TS
	linear	RLBA	Human TTR	n.d.[f]	n.d.			[23]
	branched	RLBA	Human TTR	23434[e]	0.003			[23]
		RLBA	Chicken pTTR			1330	0.02	[13]
		RLBA	Rana pTTR			2730	0.0005	[13]
		RLBA	Rana TTR			60.1[b]		[14]
		RLBA	Salmon TTR			44.3[b]		[14]
	2.4-Dinitrophenol	RLBA	Rana TTR			96.4[b]		[14]
		RLBA	Salmon TTR			91.3[b]		[14]
	4-Phenoxyphenol	RLBA	Human rTTR	n.d.	n.d.			[20]
	4-(Phenylmethyl)-phenol	RLBA	Human pTTR	>20000	<<1			TS
	4-(1,1,3,3-tetramethylbutyl)-phenol	RLBA	Human pTTR	n.d.	n.d.			TS
	2-methyl-4-(1,1,3,3-tetramethylbutyl)-phenol	RLBA	Human pTTR	n.d.	n.d.			TS
	4-tert-Pentylphenol	RLBA	Human pTTR	n.d.	n.d.			TS
	2-Chloro-4-nonylphenol	RLBA	Chicken pTTR			165	0.14	[13]
		RLBA	Rana pTTR			597	0.002	[13]

Back bone	Compound	Assay	TTR	T_4 IC_{50} (nM)	T_4 REP	T_3 IC_{50} (nM)	T_3 REP	Ref
	2,6-Dichloro-4-nonylphenol	RLBA	Chicken pTTR			7.5	3.0	[13]
		RLBA	Rana pTTR			48	0.03	[13]
	2-Chlorophenol	RLBA	Human TTR	1 000 000	0.0004			[24]
	3-Chlorophenol	RLBA	Human TTR	1 400 000	0.0003			[24]
	2.6-Dichlorophenol	RLBA	Human TTR	22 000	0.02			[24]
	2.3-Dichlorophenol	RLBA	Human TTR	130 000	0.003			[24]
	2.4.5-Trichlorophenol	RLBA	Human TTR	2700	0.15			[24]
	2.4.6-Trichlorophenol	RLBA	Human TTR	1200	0.33			[24]
	Pentachlorophenol	RLBA	Seabream rTTR	2480	0.008			[15]
		SPR	Human rTTR		0.53			[2]
		SPR	Human pTTR		0.64			[2]
		RLBA	Human pTTR	230	1.74			[24]
		RLBA	Rana pTTR			45	0.01	[14]
		RLBA	Chicken pTTR			6	3.5	[14]
		RLBA	Rana TTR			15.4[b]		[14]
		RLBA	Salmon TTR			15.1[b]		[14]
	4-OH-Heptachlorostyrene	RLBA	Human TTR	72	1.1			[22]
	2,4-Dibromphenol	RLBA	Human rTTR	1400	0.06			[20]
	2,4,6-Tribromphenol	RLBA	Human pTTR	4.8	10			[6]
		RLBA	Seabream rTTR	30	0.7			[15]
		RLBA	Human rTTR	67	1.2			[20]
		RLBA	Human rTTR	26	3			[21]
	Pentabromphenol	RLBA	Human rTTR	12	7.14			[20]
		SPR	Human rTTR		0.64			[2]
		SPR	Human pTTR		0.4			[2]
	Piperine	RLBA	Human pTTR	n.d.	n.d.			TS

Back bone	Compound	Assay	TTR	T_4 IC_{50} (nM)	T_4 REP	T_3 IC_{50} (nM)	T_3 REP	Ref
	Isosafrole	RLBA	Human pTTR	n.d.	n.d.			TS
Musks, 1 ar	Celestolide	RLBA	Human pTTR	n.d.	n.d.			TS
	Galaxolide	RLBA	Human pTTR	n.d.	n.d.			TS
	Phantolide	RLBA	Human pTTR	n.d.	n.d.			TS
	Tonalide	RLBA	Human pTTR	n.d.	n.d.			TS
	Traseolide	RLBA	Human pTTR	n.d.	n.d.			TS
	Musk ambrette	RLBA	Human pTTR	n.d.	n.d.			TS
	Musk ketone	RLBA	Human pTTR	n.d.	n.d.			TS
Biphenyl, 2 ar	Biphenyl	RLBA	Seabream rTTR	n.d.	n.d.			[15]
	CB 5	RLBA	Human TTR	87[c]				[24]
	CB 14	SPR	Human rTTR		<0.001			[2]
		SPR	Human pTTR		<0.001			[2]
		RLBA	Human TTR	11[c]				[24]
	CB 19	RLBA	Human TTR	n.d.	n.d.			[19]
	CB 28	RLBA	Human TTR	n.d.	n.d.			[19]
	CB 38	BA	Human pTTR	0.18[a]				[27]
	CB 47	RLBA	Human TTR	n.d.	n.d.			[19]
	CB 51	RLBA	Human TTR	n.d.	n.d.			[19]
	CB 52	RLBA	Human TTR	n.d.	n.d.			[19]
	CB 53	RLBA	Human TTR	n.d.	n.d.			[19]
	CB 74	RLBA	Human TTR	n.d.	n.d.			[19]
	CB 77	RLBA	Human TTR	n.d.	n.d.			[25]
	CB 80	BA	Human pTTR	0.62[a]				[27]
		RLBA	Human TTR	n.d.	n.d.			[19]
	CB 95	RLBA	Human TTR	n.d.	n.d.			[19]
	CB 100	RLBA	Human TTR	n.d.	n.d.			[19]
	CB 101	RLBA	Human TTR	n.d.	n.d.			[19]
	CB 104	RLBA	Human TTR	n.d.	n.d.			[19]

Back bone	Compound	Assay	TTR	T_4 IC_{50} (nM)	T_4 REP	T_3 IC_{50} (nM)	T_3 REP	Ref
	CB 105	RLBA	Human rTTR	> 1000	<<1			[7]
	CB 110	BA	Human pTTR	n.d.[a]				[27]
	CB 111	BA	Human pTTR	0.06[a]				[27]
	CB 118	RLBA	Human TTR	n.d.	n.d.			[19]
	CB 122	RLBA	Human TTR	790	0.083			[19]
	CB 125	RLBA	Human TTR	3700	0.014			[19]
	CB 126	RLBA	Human TTR	n.d.	n.d.			[25]
		RLBA	Human TTR	>15 000	<0.0083			[19]
	CB 127	BA	Human pTTR	1.5[a]				[27]
	CB 128	RLBA	Human TTR	n.d.	n.d.			[19]
	CB 136	RLBA	Human TTR	n.d.	n.d.			[19]
	CB 138	BA	Human pTTR	n.d.[a]				[27]
		RLBA	Human TTR	>15 000	<0.0083			[19]
	CB 153	RLBA	Human TTR	>15 000	<0.0083			[19]
	CB 162	BA	Human pTTR	0.19[a]				[27]
	CB 168	RLBA	Human TTR	1300	0.041			[19]
	CB 169	BA	Human pTTR	0.05[a]				[27]
	CB 170	RLBA	Human TTR	n.d.	n.d.			[19]
	CB 180	RLBA	Human TTR	n.d.	n.d.			[19]
	CB 187	RLBA	Gull rTTR	91	0.17	791	0.012	[8]
	CB 190	RLBA	Human TTR	n.d.	n.d.			[19]
	2’-OH CB 105	RLBA	Human rTTR	950	0.09			[7]
	3'-OH CB 138	RLBA	Human pTTR		3.3			[21]
	3'-OH CB 180	BA	Human pTTR	1.67[a]				[27]
		RLBA	Human TTR		1.9			[21]
		RLBA	Human TTR	17.8	3.4			[28]

Back bone	Compound	Assay	TTR	T_4 IC_{50} (nM)	T_4 REP	T_3 IC_{50} (nM)	T_3 REP	Ref
	3'-OH CB 182	RLBA	Human TTR	19	3.2			[28]
	3'-OH CB 30	RLBA	Human TTR	n.d.	n.d.			[25]
	3-OH CB 203	RLBA	Human TTR	44.1	1.59			[23]
	4-OH Biphenyl	RLBA	Human TTR	14[c]				[24]
	4-OH CB 14	SPR	Human rTTR		4.36			[2]
		SPR	Human pTTR		3.04			[2]
		RLBA	Human TTR	16	3.88			[25]
	4'-OH CB 35	RLBA	Human rTTR	10.5	8.4			[7]
	4'-OH CB 50	BA	Human pTTR	0.81[a]				[27]
	4-OH CB 56	RLBA	Human rTTR	8.8	10.2			[7]
	4'-OH CB 61	BA	Human pTTR	0.83[a]				[27]
		RLBA	Human TTR	89	0.7			[25]
	4'-OH CB 69	BA	Human pTTR	0.96[a]				[27]
		RLBA	Human TTR	33	1.88			[25]
	4'-OH CB 79	BA	Human pTTR	1.86[a]				[27]
		ANSA	Human TTR	510	0.52			[26]
	4'-OH CB 82	RLBA	Human rTTR	17.5	5			[7]
	4-OH CB 93	BA	Human pTTR	0.7[a]				[27]
	4'-OH CB 106	BA	Human pTTR	1.23[a]				[27]
		RLBA	Human TTR	141	0.44			[25]
	4-OH CB 107	BA	Human pTTR	1.48[a]				[27]
		RLBA	Human TTR	24.4	3.3			[18]
		RLBA	Human TTR	22.6	3.5			[21]
	4'-OH CB 108	BA	Human pTTR	1.63[a]				[27]
		RLBA	Human rTTR	15	5.9			[7]
	4'-OH CB 109	BA	Human pTTR	0.84[a]				[27]
	4’-OH CB 112	BA	Human pTTR	1.12[a]				[27]

Back bone	Compound	Assay	TTR	T_4 IC_{50} (nM)	T_4 REP	T_3 IC_{50} (nM)	T_3 REP	Ref
	4'-OH CB 118	RLBA	Human TTR	19.3	4.1			[21]
	4'-OH CB 121	RLBA	Human TTR	40	1.55			[25]
	4'-OH CB 127	RLBA	Human rTTR	10.3	8.5			[7]
	4'-OH CB 130	BA	Human pTTR	1.02[a]				[27]
		RLBA	Human TTR	21.4	3.7			[21]
	4'-OH CB 172	BA	Human pTTR	1.4[a]				[27]
		RLBA	Human pTTR	13	4.6			[28]
		RLBA	Human TTR		3.8			[21]
	4-OH-CB 187	RLBA	Gull rTTR	14	1.19	2	3.15	[8]
		RLBA	Human TTR	19.8	4			[21]
	4'-OH CB 201	RLBA	Human TTR	21	3.35			[23]
	4,4'-OH CB 80	RLBA	Human rTTR	16.5	5.4			[7]
		BA	Human pTTR	1.36[a]				[27]
		RLBA	Human TTR	11	5.6			[25]
	4,4'-OH CB 111	RLBA	Human rTTR	6.5	13.6			[7]
	4,4'-OH CB 202	RLBA	Human TTR	16.2	4.34			[23]
	5-OH CB 77	RLBA	Human rTTR	25	3.5			[7]
	5'-OH CB 105	RLBA	Human rTTR	19	4.6			[7]
	5-OH CB 183	RLBA	Human TTR	19.6	3.1			[28]
	4-MeO CB 187	RLBA	Gull rTTR	981	0.016	55	0.108	[8]
Bisphenol A, 2 ar	Bisphenol A	RLBA	Human rTTR	n.d.	n.d.			[20]
		RLBA	Seabream rTTR	n.d.	n.d.			[15]
		SPR	Human rTTR		<0.001			[2]
		SPR	Human pTTR		<0.001			[2]
		RLBA	Chicken pTTR			9070	0.002	[13]
		RLBA	Rana pTTR			1530	0.0009	[13]
		RLBA	Rana rTTR			500-700		[16]
		RLBA	Xenopus TTR			>10 000		[16]

Back bone	Compound	Assay	TTR	T_4 IC_{50} (nM)	T_4 REP	T_3 IC_{50} (nM)	T_3 REP	Ref
		RLBA	Rana pTTR			1500	0.0003	[14]
		RLBA	Chicken pTTR			6000	0.004	[14]
		RLBA	Rana TTR			19.3[b]		[14]
		RLBA	Salmon TTR			74[b]		[14]
		RLBA	Human pTTR	n.d.	n.d.			TS
	3-Chlorobisphenol A	RLBA	Chicken pTTR			367	0.06	[13]
		RLBA	Rana pTTR			173	0.008	[13]
	3,3'-Dichlorobisphenol A	RLBA	Chicken pTTR			86.3	0.26	[13]
		RLBA	Rana pTTR			18.3	0.078	[13]
	3,5-Dichlorobisphenol A	RLBA	Chicken pTTR			19	1.18	[13]
		RLBA	Rana pTTR			44.7	0.032	[13]
	3,3',5-Trichlorobisphenol A	RLBA	Chicken pTTR			33.7	0.67	[13]
		RLBA	Rana pTTR			10.8	0.13	[13]
	3,3',5,5'-Tetrachlorobisphenol A	RLBA	Chicken pTTR			22.7	0.99	[13]
		RLBA	Rana pTTR			18.7	0.076	[13]
		RLBA	Human rTTR	107	0.76			[20]
		SPR	Human rTTR		0.75			[2]
		SPR	Human pTTR		0.66			[2]
	Bisphenol A bis (2,3-dihydroxypropyl) ether	RLBA	Human rTTR	n.d.	n.d.			[20]
	Bisphenol A bis (3-chloro-2-hydroxypropyl) ether	RLBA	Human rTTR	n.d.	n.d.			[20]
	Bisphenol A diglycidyl ether	RLBA	Human rTTR	n.d.	n.d.			[20]
	Bisphenol A diglycidyl ether, brominated	RLBA	Human rTTR	n.d.	n.d.			[20]

Back bone	Compound	Assay	TTR	T_4 IC_{50} (nM)	T_4 REP	T_3 IC_{50} (nM)	T_3 REP	Ref
	Monobromobisphenol A	RLBA	Human rTTR	n.d.	n.d.			[20]
	Dibromo bisphenol A	RLBA	Human rTTR	n.d.	n.d.			[20]
	Tribromobisphenol A	RLBA	Human rTTR	140	0.58			[20]
	Tetrabromo bisphenol A (TBBPA)	RLBA	Human pTTR	31	1.6			[6]
		RLBA	Seabream rTTR	2.1	10			[15]
		RLBA	Human rTTR	7.7	10.6			[20]
		SPR	Human rTTR		1.5			[2]
		SPR	Human pTTR		1.06			[2]
	TBBPA-DBPE	RLBA	Human pTTR	5200	0.0086			[6]
		RLBA	Seabream rTTR	n.d.	n.d.			[15]
Diphenyl ether, 2 ar	Pentamix PBDE	RLBA	Seabream rTTR	8.3	2.5			[15]
		RLBA	Human pTTR	n.d.	n.d.			[6]
	Octamix PBDE	RLBA	Human pTTR	n.d.	n.d.			[6]
	BDE 19	RLBA	Human pTTR	n.d.	n.d.			[6]
		RLBA	Seabream rTTR	44	0.5			[15]
	BDE 28	RLBA	Human pTTR	n.d.	n.d.			[6]
		RLBA	Seabream rTTR	14.9	1.4			[15]
	BDE 38	RLBA	Human pTTR	>25 000	n.d.			[6]
		RLBA	Seabream rTTR	65	0.3			[15]
	BDE 39	RLBA	Human pTTR	n.d.	n.d.			[6]
		RLBA	Seabream rTTR	9.5	2.2			[15]
	BDE 47	RLBA	Human pTTR	>25 000	n.d.			[6]
		RLBA	Human rTTR	36 000	0.0025			[17]
		RLBA	Seabream rTTR	5.3	4			[15]
		RLBA	Gull rTTR	89	0.176	529	0.011	[8]
	BDE 49	RLBA	Human pTTR	>25 000	n.d.			[6]
		RLBA	Seabream rTTR	0.5	42			[15]

Back bone	Compound	Assay	TTR	T_4 IC_{50} (nM)	T_4 REP	T_3 IC_{50} (nM)	T_3 REP	Ref
	BDE 79	RLBA	Human pTTR	n.d.	n.d.			[6]
	BDE 99	RLBA	Human pTTR	n.d.	n.d.			[6]
		RLBA	Seabream rTTR	6.7	3.1			[15]
	BDE 100	RLBA	Human pTTR	n.d.	n.d.			[6]
		RLBA	Seabream rTTR	56	0.4			[15]
	BDE 127	RLBA	Human pTTR	22 000	0.0025			[6]
		RLBA	Seabream rTTR	n.d.	n.d.			[15]
	BDE 153	RLBA	Human pTTR	n.d.	n.d.			[6]
		RLBA	Seabream rTTR	n.d.	n.d.			[15]
	BDE 155	RLBA	Human pTTR	n.d.	n.d.			[6]
		RLBA	Seabream rTTR	n.d.	n.d.			[15]
	BDE 169	RLBA	Human pTTR	n.d.	n.d.			[6]
		RLBA	Seabream rTTR	n.d.	n.d.			[15]
	BDE 181	RLBA	Human pTTR	7000	0.01			[6]
		RLBA	Seabream rTTR	n.d.	n.d.			[15]
	BDE 183	RLBA	Human pTTR	n.d.	n.d.			[6]
		RLBA	Seabream rTTR	n.d.	n.d.			[15]
	BDE 185	RLBA	Human pTTR	7400	0.01			[6]
		RLBA	Seabream rTTR	n.d.	n.d.			[15]
	BDE 190	RLBA	Human pTTR	9000	0.008			[6]
		RLBA	Seabream rTTR	n.d.	n.d.			[15]
	BDE 206	RLBA	Human pTTR	n.d.	n.d.			[6]
		RLBA	Seabream rTTR	n.d.	n.d.			[15]
	BDE 209	RLBA	Human pTTR	n.d.	n.d.			[6]
		RLBA	Seabream rTTR	n.d.	n.d.			[15]
	2'-OH BDE 3	BA	Human TTR	200	0.15		1.4	[3]
		FLU-TTR	Human TTR	4132	0.06			[4]
	2'-OH BDE 7	BA	Human TTR	100	0.3		2.8	[3]

Back bone	Compound	Assay	TTR	T_4 IC_{50} (nM)	T_4 REP	T_3 IC_{50} (nM)	T_3 REP	Ref
		FLU-TTR	Human TTR	864	0.3			[4]
	2'-OH BDE 28	BA	Human TTR	10	3.0		28	[3]
		RLBA	Human rTTR	n.d.	n.d.			[20]
		FLU-TTR	Human TTR	900	0.29			[4]
	2'-OH BDE-66	RLBA	Human TTR	170	0.65			[17]
		ANSA	Human TTR	230	0.93			[26]
	2'-OH BDE 68	BA	Human TTR	8	3.75		35	[3]
	3'-OH BDE 7	BA	Human TTR	50	0.6		5.6	[3]
		FLU-TTR	Human TTR	375	0.69			[4]
	3'-OH BDE 28	BA	Human TTR	30	1.0		9.3	[3]
		FLU-TTR	Human TTR	360	0.72			[4]
	3-OH BDE 47	RLBA	Human TTR	17	4.0			[17]
		BA	Human TTR	7	4.3		40	[3]
		FLU-TTR	Human TTR	110	2.36			[4]
	3-OH BDE 100	FLU-TTR	Human TTR	219	1.19			[4]
	3-OH BDE 154	BA	Human TTR	10	3.0		28	[3]
		FLU-TTR	Human TTR	200	1.3			[4]
	4'-OH BDE 17	BA	Human TTR	20	1.5		14	[3]
	4-OH BDE 42	RLBA	Human TTR	19	3.5			[17]
		BA	Human TTR	9	3.3		31	[3]
		ANSA	Human TTR	290	0.93			[26]
	4'-OH BDE 49	RLBA	Human TTR	19	3.5			[17]
		RLBA	Gull rTTR	7.7	2.05	4.89	1.12	[8]
		BA	Human TTR	10	3.0		28	[3]
	4-OH BDE 188	FLU-TTR	Human TTR	190	1.37			[4]
	5-OH BDE 47	RLBA	Human TTR	25	3.0			[17]
		BA	Human TTR	4	7.5		70	[3]
		FLU-TTR	Human TTR	218	1.19			[4]

Back bone	Compound	Assay	TTR	T_4 IC_{50} (nM)	T_4 REP	T_3 IC_{50} (nM)	T_3 REP	Ref
	6-OH BDE 47	RLBA	Human rTTR	180	0.26			[6]
		RLBA	Human TTR	150	0.39			[17]
		RLBA	Seabream rTTR	700	0.03			[15]
		RLBA	Gull rTTR	11.9	1.32	69	0.07	[8]
		BA	Human TTR	15	2.0		19	[3]
		ANSA	Human TTR	630	0.42			[26]
		FLU-TTR	Human TTR	323	0.81			[4]
	6-OH BDE 85	BA	Human TTR	7	4.3		40	[3]
	6-MeO BDE 47	RLBA	Gull rTTR	54.3	0.29	234	0.025	[8]
	Triclosan	RLBA	Human pTTR	4179	0.015			TS
	2-Bromo-4-(2,4,6-tribromo-phenoxy) phenol	RLBA	Human rTTR	66	1.22			[20]
	2.6-Dibromo-4-(2,4,6-tribromo-phenoxy) phenol	RLBA	Human rTTR	57	1.42			[20]
	4-(2,4,6-Tribromo-phenoxy) phenol	RLBA	Human rTTR	199	0.41			[20]
Diphenyl ethan, 2 ar	o,p'-DDT	RLBA	Human TTR	n.d.	n.d.			[25]
	o,p'-DDD	RLBA	Human TTR	n.d.	n.d.			[25]
	p,p'-DDOH	RLBA	Human TTR	n.d.	n.d.			[25]
	p,p'-DDE	RLBA	Human TTR	n.d.	n.d.			[25]
	p,p'-DDT	RLBA	Human TTR	n.d.	n.d.			[25]
	Dicofol (Kelthane)	RLBA	Chicken pTTR			5000	0.004	[14]
		RLBA	Rana TTR			196.3[b]		[14]
		RLBA	Salmon TTR			96.7[b]		[14]
	Hexachlorophene	RLBA	Human TTR	600	0.67			[24]
Stilbene, 2 ar	Diethylstilbestrol	RLBA	Seabream rTTR	4850	0.004			[15]
		RLBA	Rana rTTR			9		[16]
		RLBA	Xenopus TTR			400-550		[16]

Back bone	Compound	Assay	TTR	T_4 IC_{50} (nM)	T_4 REP	T_3 IC_{50} (nM)	T_3 REP	Ref
		RLBA	Rana pTTR			0.3	1.7	[14]
		RLBA	Chicken pTTR			0.4	53	[14]
		RLBA	Rana TTR			4.1[b]		[14]
		RLBA	Salmon TTR			7.4[b]		[14]
Dioxin, 2 ar	2-OH-1,37,8-CDD	RLBA	Human rTTR	31.6	4.37			[7]
	7-OH-2,3,8-CDD	RLBA	Human rTTR	136	1			[7]
	8-OH-2,3-CDD	RLBA	Human rTTR	>1000	<<1			[7]
Furan, 2 ar	2-OH-7,8-CDF	RLBA	Human rTTR	>1000	<1			[7]
	3-OH-2,6,7,8-CDF	RLBA	Human rTTR	30.2	4.5			[7]
	8-OH-2,3,4-CDF	RLBA	Human rTTR	>1000	<<1			[7]
Other, 2 ar	Diphenyl sulfone	RLBA	Human pTTR	n.d.	n.d.			TS
	(2E)-1,4-Diphenyl-2-butene-1,4-dione	RLBA	Human pTTR	n.d.	n.d.			TS
	Diclofenac	RLBA	Human pTTR	2200	0.032			TS
	Triclocarban	RLBA	Human pTTR	n.d.	n.d.			TS
PAH, >2 ar	Perinaphthenone	RLBA	Human pTTR	n.d.	n.d.			TS
	Aminopyrene	RLBA	Rana TTR			69.4[b]		[14]
		RLBA	Salmon TTR			43.5[b]		[14]
	Benzo(a)pyrene	RLBA	Rana TTR			96[b]		[14]
		RLBA	Salmon TTR			96.6[b]		[14]
	7H-Benz[de]anthracen-7-onea	RLBA	Human pTTR	n.d.	n.d.			TS
Organo phosphate	Tris(2-chloro-iso-propyl) phosphate	RLBA	Human pTTR	n.d.	n.d.			TS
	Tris(2-chloro-ethyl) phosphate	RLBA	Human pTTR	n.d.	n.d.			TS
	Triphenyl phosphate	RLBA	Human pTTR	n.d.	n.d.			TS
	Tris(2-ethylhexyl) phosphate	RLBA	Human pTTR	n.d.	n.d.			TS

Back bone	Compound	Assay	TTR	T_4 IC_{50} (nM)	T_4 REP	T_3 IC_{50} (nM)	T_3 REP	Ref
	Cresyl diphenyl phosphate	RLBA	Human pTTR	n.d.	n.d.			TS
Phthalate	Butylbenzyl phthalate	RLBA	Rana TTR			75.4[b]		[14]
		RLBA	Salmon TTR			78.9[b]		[14]
		RLBA	Human pTTR	n.d.	n.d.			TS
	Di-2-ethylhexyl phthalate	RLBA	Rana TTR			97.6[b]		[14]
		RLBA	Salmon TTR			82.8[b]		[14]
	Di-n-butyl phthalate	RLBA	Rana TTR			89.2[b]		[14]
		RLBA	Salmon TTR			85.8[b]		[14]
		RLBA	Human pTTR	n.d.	n.d.			TS
Cyclo-alkane	Mirex	RLBA	Rana TTR			30.1[b]		[14]
		RLBA	Salmon TTR			92.4[b]		[14]
	alpha-HBCDD	RLBA	Human rTTR	12 000	0.0027			[6]
		RLBA	Seabream rTTR	n.d.	n.d.			[15]
	beta-HBCDD	RLBA	Human rTTR	25 000	0.0023			[6]
		RLBA	Seabream rTTR	n.d.	n.d.			[15]
	gamma-HBCDD	RLBA	Human rTTR	n.d.				[6]
		RLBA	Seabream rTTR	n.d.	n.d.			[15]
	HBCDD technical mixture	RLBA	Human rTTR	n.d.				[6]
	Retinoic acid	RLBA	Rana rTTR			>50 000		[16]
		RLBA	Xenopus TTR			>50 000		[16]
	Retinol	RLBA	Rana rTTR			>50 000		[16]
		RLBA	Xenopus TTR			>50 000		[16]
	Cholesterol	ANSA	Human TTR	> 30 000	<0.01			[26]
	Naphthenic acid	RLBA	Human pTTR	n.d.	n.d.			TS
Alkane	Hexaethyleneglycol monododecyl ether	RLBA	Human rTTR	n.d.	n.d.			TS

Back bone	Compound	Assay	TTR	T_4 IC_{50} (nM)	T_4 REP	T_3 IC_{50} (nM)	T_3 REP	Ref
	Malathion	RLBA	Rana pTTR			27 000	<<	[14]
		RLBA	Chicken pTTR			900	0.02	[14]
		RLBA	Rana TTR			87[b]		[14]
		RLBA	Salmon TTR			91.7[b]		[14]
	Methoprene	RLBA	Human TTR	n.d.				[25]
	Squalene	RLBA	Human pTTR	n.d.	n.d.			TS
	Hexanoic acid	RLBA	Human rTTR	n.d.	n.d.			[9]
	Octanoic acid	RLBA	Human rTTR	n.d.	n.d.			[9]
	Decanoic acid	RLBA	Human rTTR	n.d.	n.d.			[9]
	Dodecanoic acid	ANSA	Human TTR	11 000	0.02			[26]
		RLBA	Human rTTR	n.d.	n.d.			[9]
	Myristic acid	ANSA	Human TTR	6500	0.04			[26]
		RLBA	Human rTTR	n.d.	n.d.			[9]
	Palmitic acid	ANSA	Human TTR	n.d.	0.12[g]			[26]
	Stearic acid	ANSA	Human TTR	> 300 000	<0.001			[26]
		RLBA	Human rTTR	n.d.	n.d.			[9]
	Arachidonic acid	ANSA	Human TTR	2300	0.12			[26]
	Linoleic acid	ANSA	Human TTR	6500	0.04			[26]
	Oleic acid	ANSA	Human TTR	3300	0.08			[26]
	PFBA	RLBA	Human rTTR	n.d.	n.d.			[9]
	PFPeA	RLBA	Human rTTR	439	0.18			[21]
	PFHxA	RLBA	Human rTTR	8220	0.007			[9]
	PFHpA	RLBA	Human rTTR	1565	0.039			[9]
	PFOA	RLBA	Human rTTR	949	0.064			[9]
	PFNA	RLBA	Human rTTR	2737	0.022			[9]
	PFDcA	RLBA	Human rTTR	8954	0.007			[9]
	PFUnA	RLBA	Human rTTR	21560	0.003			[9]

Back bone	Compound	Assay	TTR	T_4 IC_{50} (nM)	T_4 REP	T_3 IC_{50} (nM)	T_3 REP	Ref
	PFDoA	RLBA	Human rTTR	46894	0.001			[9]
	PFTrDA	RLBA	Human rTTR	1234	0.064			[21]
	PFTedA	RLBA	Human rTTR	28996	0.002			[9]
	DoFHpA	RLBA	Human rTTR	8637	0.007			[9]
	FHUEA	RLBA	Human rTTR	8848	0.007			[9]
	PFBS	RLBA	Human rTTR	19460	0.003			[9]
	PFHxS	RLBA	Human rTTR	717	0.085			[9]
	PFOS	RLBA	Human rTTR	940	0.065			[9]
	L-PFDS	RLBA	Human rTTR	n.d.	n.d.			[9]
	L-PFOSi	RLBA	Human rTTR	1733	0.035			[9]
	FTSA (6:2)	RLBA	Human rTTR	n.d.	n.d.			[21]
	FTOH (6:2)	RLBA	Human rTTR	n.d.	n.d.			[9]
	FTOH (8:2)	RLBA	Human rTTR	n.d.	n.d.			[9]
	N-MeFOSE	RLBA	Human rTTR	n.d.	n.d.			[9]
	N-EtFOSE	RLBA	Human rTTR	n.d.	n.d.			[9]
	FOSA	RLBA	Human rTTR	6124	0.01			[9]
	N,N-Me3FOSA	RLBA	Human rTTR	n.d.	n.d.			[9]
	N-MeFOSA	RLBA	Human rTTR	n.d.	n.d.			[9]
	N-EtFOSA	RLBA	Human rTTR	n.d.	n.d.			[9]

n.d. = not detected
TS = This Study
RLBA = Radio Ligand Binding Assay
BA = Binding Assay
SPR = Surface Plasmon Resonance based assay
ANSA = ANSA displacement assay based on the salt 8-anilino-1-naphthalenesulfonic acid ammonium
FLU-TTR = A fluorescein−thyroxine (F−T_4) conjugate assay
[a] Antibody based binding assay with relative binding potencies in plasma (<0.2 is low binding affinity and >0.7 is high affinity).
[b] Effects (%) of competitor (8 000nM) on [^{125}I]T3 binding to chicken and salmon TTR. <90% is significant binding competition.
[c] Competition (%) of competitor (100 000nM) on [^{125}I]T_4 binding to human. the higher percentage the more potent competitor.
[d] Technical mixture CAS 25154-52-3
[e] Branched CAS 84852-15-3
[f] Linear CAS 104-40-5.
[g] Calculated on IC20 value

Table S2. Brief explanation of the chemical descriptors calculated using the Molecular Operating Environment software

Descriptor	Description
VDistEq	If *m* is the sum of the distance matrix entries then VdistEq is defined to be the sum of $\log_2 m - p_i \log_2 p_i / m$ where p_i is the number of distance matrix entries equal to *i*.
VDistMa	If *m* is the sum of the distance matrix entries then VDistMa is defined to be the sum of $\log_2 m - D_{ij} \log_2 D_{ij} / m$ over all *i* and *j*.
b_1rotR	Fraction of rotatable single bonds: b_1rotN divided by b_heavy.
Weight	Molecular weight (including implicit hydrogens) with atomic weights taken from [CRC 1994].
chi0	Atomic connectivity index (order 0) from [Hall 1991] and [Hall 1977]. this is calculated as the sum of 1/sqrt(d_i) over all heavy atoms *i* with $d_i > 0$.
chi1	Atomic connectivity index (order 1) from [Hall 1991] and [Hall 1977]. this is calculated as the sum of 1/sqrt($d_i d_j$) over all bonds between heavy atoms *i* and *j* where $i < j$.
VAdjEq	Vertex adjacency information (equality): $-(1-f)\log_2(1-f) - f \log_2 f$ where $f = (n^2 - m) / n^2$, *n* is the number of heavy atoms and *m* is the number of heavy-heavy bonds. If *f* is not in the open interval (0,1), then 0 is returned.
VAdjMa	Vertex adjacency information (magnitude): $1 + \log_2 m$ where *m* is the number of heavy-heavy bonds. If *m* is zero, then zero is returned.
balabanJ	Balaban's connectivity topological index [Balaban 1982].
PEOE_PC+	Total positive partial charge: the sum of the positive qi. Q_PC+ is identical to PC+ which has been retained for compatibility.
PEOE_PC-	Total negative partial charge: the sum of the negative q_i. Q_PC- is identical to PC- which has been retained for compatibility.
PEOE_RPC+	Relative positive partial charge: the largest positive q_i divided by the sum of the positive q_i. Q_RPC+ is identical to RPC+ which has been retained for compatibility.
PEOE_RPC-	Relative negative partial charge: the smallest negative q_i divided by the sum of the negative q_i. Q_RPC- is identical to RPC- which has been retained for compatibility.
PEOE_VSA_FHYD	Fractional hydrophobic Van der Waals surface area. this is the sum of the v_i such that $\|q_i\|$ is less than or equal to 0.2 divided by the total surface area. the v_i are calculated using a connection table approximation.
PEOE_VSA_FNEG	Fractional negative Van der Waals surface area. this is the sum of the v_i such that q_i is negative divided by the total surface area. the v_i are calculated using a connection table approximation.
PEOE_VSA_FPNEG	Fractional negative polar Van der Waals surface area. this is the

	sum of the v_i such that q_i is less than -0.2 divided by the total surface area. the v_i are calculated using a connection table approximation.
PEOE_VSA_FPOL	Fractional polar Van der Waals surface area. this is the sum of the v_i such that $\|q_i\|$ is greater than 0.2 divided by the total surface area. the v_i are calculated using a connection table approximation.
PEOE_VSA_FPOS	Fractional positive Van der Waals surface area. this is the sum of the v_i such that q_i is non-negative divided by the total surface area. the v_i are calculated using a connection table approximation.
PEOE_VSA_FPPOS	Fractional positive polar Van der Waals surface area. this is the sum of the v_i such that q_i is greater than 0.2 divided by the total surface area. the v_i are calculated using a connection table approximation.
PEOE_VSA_HYD	Total hydrophobic Van der Waals surface area. this is the sum of the v_i such that $\|q_i\|$ is less than or equal to 0.2. the v_i are calculated using a connection table approximation.
PEOE_VSA_NEG	Total negative Van der Waals surface area. this is the sum of the v_i such that q_i is negative. the v_i are calculated using a connection table approximation.
PEOE_VSA_PNEG	Total negative polar Van der Waals surface area. this is the sum of the v_i such that q_i is less than -0.2. the v_i are calculated using a connection table approximation.
PEOE_VSA_POL	Total polar Van der Waals surface area. this is the sum of the v_i such that $\|q_i\|$ is greater than 0.2. the v_i are calculated using a connection table approximation.
PEOE_VSA_POS	Total positive Van der Waals surface area. this is the sum of the v_i such that q_i is non-negative. the v_i are calculated using a connection table approximation.
PEOE_VSA_PPOS	Total positive polar Van der Waals surface area. this is the sum of the v_i such that q_i is greater than 0.2. the v_i are calculated using a connection table approximation.
Kier1	First kappa shape index: $(n\text{-}1)^2 / m^2$ [Hall 1991].
Kier2	Second kappa shape index: $(n\text{-}1)^2 / m^2$ [Hall 1991].
Kier3	third kappa shape index: $(n\text{-}1)\ (n\text{-}3)^2 / p_3^2$ for odd n, and $(n\text{-}3)\ (n\text{-}2)^2 / p_3^2$ for even n [Hall 1991].
KierFlex	Kier molecular flexibility index: (KierA1) (KierA2) / n [Hall 1991].
logS	Log of the aqueous solubility this property is calculated from an atom contribution linear atom type model [Hou 2004] with r^2 = 0.90, ~1,200 molecules.
apol	Sum of the atomic polarizabilities (including implicit hydrogens) with polarizabilities taken from [CRC 1994].
bpol	Sum of the absolute value of the difference between atomic polarizabilities of all bonded atoms in the molecule (including implicit hydrogens) with polarizabilities taken from [CRC 1994].

mr	Molecular refractivity (including implicit hydrogens). this property is calculated from an 11 descriptor linear model [MREF 1998] with r^2 = 0.997, RMSE = 0.168 on 1,947 small molecules.
SMR	Molecular refractivity (including implicit hydrogens). this property is an atomic contribution model [Crippen 1999] that assumes the correct protonation state (washed structures). the model was trained on ~7000 structures and results may vary from the mr descriptor.
TPSA	Polar surface area calculated using group contributions to approximate the polar surface area from connection table information only. the parameterization is that of Ertl *et al.* [Ertl 2000].
density	Molecular mass density: Weight divided by vdw_vol.
vdw_area	Area of Van der Waals surface calculated using a connection table approximation.
vdw_vol	Van der Waals volume calculated using a connection table approximation.
logP(o/w)	Log of the octanol/water partition coefficient (including implicit hydrogens). this property is calculated from a linear atom type model [LOGP 1998] with r^2 = 0.931, RMSE=0.393 on 1,827 molecules.
diameter	Largest value in the distance matrix [Petitjean 1992].
radius	If r_i is the largest matrix entry in row i of the distance matrix D, then the radius is defined as the smallest of the r_i [Petitjean 1992].
wienerPath	Wiener path number: half the sum of all the distance matrix entries as defined in [Balaban 1979] and [Wiener 1947].
wienerPol	Wiener polarity number: half the sum of all the distance matrix entries with a value of 3 as defined in [Balaban 1979].
a_aro	Number of aromatic atoms.
b_1rotN	Number of rotatable single bonds. Conjugated single bonds are not included (e.g., ester and peptide bonds).
b_ar	Number of aromatic bonds.
b_double	Number of double bonds. Aromatic bonds are not considered to be double bonds.
rings	Number of rings.
zagreb	Zagreb index: the sum of d_i^2 over all heavy atoms i.
b_double/b_count	Number of double bonds. / Number of bonds (including implicit hydrogens). this is calculated as the sum of (di/2 + hi) over all non-trivial atoms i.
b_ar/b_count	Number of aromatic bonds / Number of bonds
b_single/b_count	Number of single bonds / Number of bonds
a_aro/a_count	Number of aromatic atoms / Number of atoms
a_don/a_count	Number of hydrogen bond donor atoms / Number of atoms
a_acc/a_count	Number of hydrogen bond acceptor atoms (not counting acidic atoms but counting atoms that are both hydrogen bond donors

	and acceptors such as -OH). / Number of atoms
a_hyd / a_count	Number of hydrophobic atoms. / Number of atoms
rings/a_count	the number of rings. / Number of atoms

Fig. S1. Loading plot with first (p[1]) versus second (p[2]) loading vector from the PCA including the 186 compounds with data from the human TTR assay. Variables are abbreviated according to Table S2

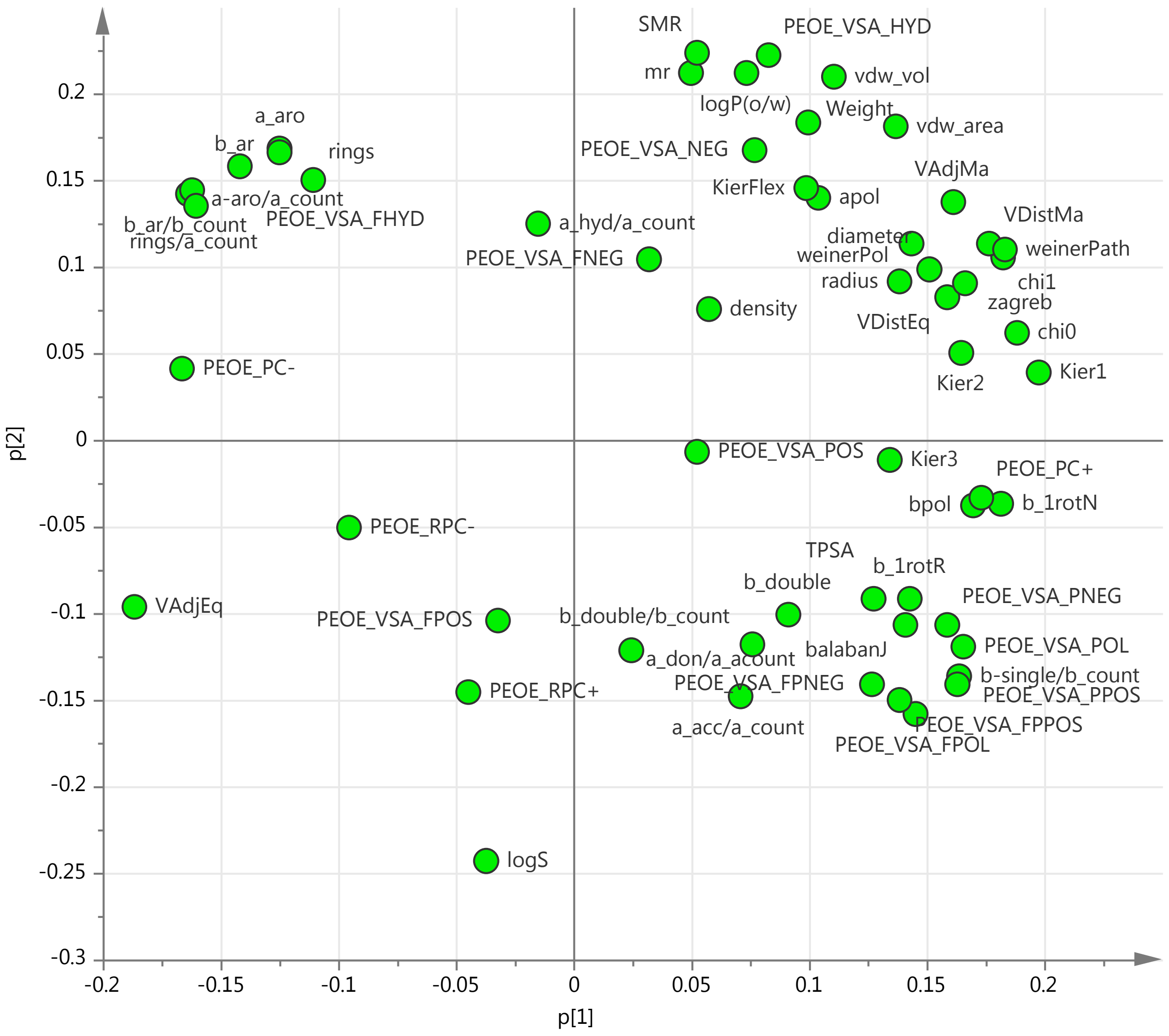

References

1. Purkey HE, Dorrel MI, Kelly JK (2001) Evaluating the binding selectivity of transthyretin amyloid fibril inhibitors in blood plasma. PNAS 98 (10):5566-5571

2. Marchesini GR, Meulenberg EP, Haasnoot W, Mizuguchi M, Irth H (2006) Biosensor recognition of thyroid-disrupting chemicals using transport proteins. Anal Chem 78:1107-1114

3. Cao J, Lin Y, Guo L-H, Zhang A-Q, Wei Y, Yang Y (2010) Structure-based investigation on the binding interaction of hydroxylated polybrominated diphenyl ethers with thyroxine transport proteins. Toxicol 277 (1-3):20-28

4. Ren XM, Guo L-H (2012) Assessment of the binding of hydroxylated polybrominated diphenyl ethers to thyroid hormone transport proteins using a site-specific fluorescence probe. Environ Sci Technol 46:4633-4640

5. Somack R, Nordeen SK, Eberhardt NL (1982) Photoaffinity labeling of human thyroxine-binding prealbumin with thyroxine and N-(ethyl-2-diazomalonyl)thyroxine. Biochem 21:5651-5660

6. Hamers T, Kamstra JH, Sonneveld E, Murk AJ, Kester MHA, Andersson PL, Legler J, Brouwer A (2006) *In vitro* profiling of the endocrine-disrupting potency of brominated flame retardants. Toxicol Sci 92 (1):157-173

7. Lans MC, Klasson-Wheler E, Willemsen M, Meussen E, Safe S, Brouwer A (1993) Structure-dependent, competetive interaction of hydroxy-polychlorobiphenyls, -dibenzo-*p*-dioxins and -dibenzofurans with human transthyretin. Chem- Biol Interactions 88:7-21

8. Ucan-Marin F, Arukwe A, Mortensen A, Gabrielsen GW, Letcher RJ (2010) Recombinant albumin transport proteins from two gull species and human: chlorinated and brominated contaminant binding and thyroid hormones. Environ Sci Technol 44 (1):497-504

9. Weiss JM, Andersson PL, Lamoree MH, Leonards PEG, van Leeuwen SPJ, Hamers T (2009) Competitive binding of perfluorinated compounds to the thyroxine transport protein transthyretin. Toxicol Sci 109 (2):206-216

10. Lübke-von Varel U, Machala M, Ciganek M, Neca J, Pencikova K, Palkova L, Vondracek J, Löffler I, Streck G, Reifferscheid G, Flückiger-Isler S, Weiss JM, Lamoree MH, Brack W (2011) Polar compounds dominate *in vitro* effects of sediment extracts. Environ Sci Technol 45:2384-2390

11. Houtman CJ, Cenijn PH, Hamers T, Lamoree MH, Legler J, Murk AJ, Brouwer A (2004) Toxicological profiling of sediment using *in vitro* bioassays, with emphasis on endocrine disruption. Environ Toxicol Chem 23 (1):32-40
12. Bytingsvik J, Simon E, Leonards PEG, Lamoree M, Lie E, Aars J, Derocher AE, Wiig Ø, Jenssen BM, Hamers T (2013) Transthyretin-binding activity of contaminants in blood from polar bear (*Ursus maritimus*) cubs. Environ Sci Technol 47 (9):4778-4786. doi:10.1021/es305160v
13. Yamauchi K, Ishihara A, Fukazawa H, Terao Y (2003) Competitive interactions of chlorinated phenol compounds with 3,3',5-triiodothyronine binding to transthyretin: detection of possible thyroid-disrupting chemicals in environmental waste water. Toxicol Appl Pharmacol 187:110-117
14. Ishihara A, Sawatsubashi S, Yamauchi K (2003) Endocrine disrupting chemicals: interference of thyroid hormone binding to transthyretins and to thyroid hormone receptors. Mol Nutr Food Res 199:105-117
15. Morgado I, Hamers T, van der Veen L, Power DM (2007) Disruption of thyroid hormone binding to sea bream recombinant transthyretin by ioxinyl and polybrominated diphenyl ethers. Chemosphere 69:155-163
16. Yamauchi K, Prapunpoj P, Richardson SJ (2000) Effect of diethylstilbestrol on thyroid hormone binding to amphibian transthyretins. Gen Comp Endocrin 119:329-339
17. Hamers T, Kamstra JH, Sonneveld E, Murk AJ, Visser TJ, van Velzen MJM, Brouwer A, Bergman Å (2008) Biotransformation of brominated flame retardants into potentially endocrine-disrupting metabolites, with special attention to 2,2',4,4'-tetrabromodiphenyl ether (BDE-47). Mol Nutr Food Res 52 (2):284-298
18. Meerts IATM, Assink Y, Cenijn PH, Van den Berg JHJ, Weijers BM, Bergman Å, Koeman JH, Brouwer A (2002) Placental transfer of a hydroxylated polychlorinated biphenyl and effects on fetal and maternal thyroid hormone homeostasis in the rat. Toxicol Sci 68:361-371
19. Hamers T, Kamstra JH, Cenijn PH, Pencikova K, Palkova L, Simeckova P, Vondracek J, Andersson PL, Stenberg M, Machala M (2011) *In vitro* toxicity profiling of ultrapure non-dioxin-like polychlorinated biphenyl congeners and their relative toxic contribution to PCB mixtures in humans. Toxicol Sci 121 (1):88-100
20. Meerts IATM, van Zanden JJ, Luijks EAC, van Leeuwen-Bol I, Marsh G, Jakobsson E, Bergman Å, Brouwer A (2000) Potent competitive interactions of some

brominated flame retardants and related compounds with human transthyretin *in vitro* Toxicol Sci 56:95-104
21. Simon E, Bytingsvik J, Jonker W, Leonards PEG, De Boer J, Jenssen BM, Lie E, Aars J, Hamers T, Lamoree MH (2011) Blood plasma sample preparation method for the assessment of thyroid hormone-disrupting potency in effect-directed analysis. Environ Sci Technol 45:7936-7944
22. Sandau CD, Meerts IATM, Letcher RJ, McAlees AJ, Chittim B, Brouwer A, Norstrom RJ (2000) Identification of 4-hydroxyheptachlorostyrene in polar bear plasma and its binding affinity to transthyretin: a metabolite of octachlorostyrene? Environ Sci Technol 34 (18):3871-3877
23. Simon E, van Velzen M, Brandsma SH, Lie E, Løken K, de Boer J, Bytingsvik J, Jenssen BM, Aars J, Hamers T, Lamoree MH (2013) Effect-directed analysis to explore the polar bear exposome: identification of thyroid hormone disrupting compounds in plasma. Environ Sci Technol 47 (15):8902-8912
24. van den Berg KJ (1990) Interactions of chlorinated phenols with thyroxine binding sites of human transthyretin, albumin and thyroid binding globulin. Chem- Biol Interactions 76:63-75
25. Cheek AO, Kow K, Chen J, McLachlan JA (1999) Potential mechanisms of thyroid disruption in humans: Interaction of organochlorine compounds with thyroid receptor, transthyretin, and thyroid-binding globulin. Environ Health Perspect 107 (4):273-278
26. Montaño M, Cocco E, Guignard C, Marsh G, Hoffmann L, Bergman Å, Gutleb AC, Murk AJ (2012) New approaches to assess the transthyretin binding capacity of bioactivated thyroid hormone disruptors. Toxicol Sci 130 (1):94-105
27. Purkey H, Palaninathan SK, Kent KC, Smith C, Safe SH, Sacchettini JC, Kelly JW (2004) Hydroxylated polychlorinated biphenyls selectively bind transthyretin in blood and inhibit amyloidogenesis: rationalizing rodent PCB toxicity. Chem Biol 11 (12):1719-1728
28. Viluksela M, Heikkinen P, Van der Ven LTM, Rendel F, Roos R, Esteban J, Korkalainen M, Lensu S, Miettinen HM, Savolainen K, Sankari S, Lilienthal H, Adamsson A, Toppari J, Herlin M, Finnilä M, Tuukkanen J, Leslie HA, Hamers T, Hamscher G, Al-Anati L, Stenius U, Dervola KS, Bogen IL, Fonnum F, Andersson PL, Schrenk D, Halldin K, Håkansson H (2014) Toxicological profile of ultrapure 2,2',3,4,4',5,5'-heptachlorbiphenyl (PCB 180) in adult rats. PLoS ONE 9: e104639
